# Predicting global and regional respiratory response in ARDS patients through individualized physics-based computational lung models: a prospective clinical pilot study

**DOI:** 10.1186/s12931-026-03949-z

**Published:** 2026-10-17

**Authors:** Armin Sablewski, Maximilian Ludwig, Carolin Eichinger, Matthias Lindner, Inéz Frerichs, Patrick Langguth, Dirk Schädler, Wolfgang A. Wall, Tobias Becher

**Affiliations:** 1 Department of Anesthesiology and Intensive Care Medicine, University Medical Center Schleswig-Holstein, Campus Kiel, Kiel, Germany; 2 Institute for Computational Mechanics, Technical University of Munich, Garching b. München, Germany; 3 Department of Radiology and Neuroradiology, University Medical Center Schleswig-Holstein, Campus Kiel, Kiel, Germany; 4 Ebenbuild GmbH, Munich, Germany

**Keywords:** lung modeling, Electrical impedance tomography, Personalized mechanical ventilation

## Abstract

**Background:**

Lung-protective ventilation is essential in the management of acute respiratory distress syndrome (ARDS), but current approaches are limited by the heterogeneity of lung pathology and a lack of individualized strategies. Patient-specific computational models may overcome these limitations by providing predictive and physiologically interpretable insights into global and regional lung behavior.

**Methods:**

Ten mechanically ventilated patients with ARDS were evaluated in a monocentric prospective pilot study. A predefined study protocol included ventilatory maneuvers with systematic changes in ventilator mode, tidal volume, positive end-expiratory pressure (PEEP), and driving pressure. For each patient, a personalized, regionally resolved physics-based computational lung model was generated and calibrated to reproduce the individual respiratory system mechanics. Model predictions of tidal volume, airway and transpulmonary pressures, end-expiratory lung volume (EELV), and electrical impedance tomography (EIT)-derived regional ventilation distribution were simulated for different PEEP levels and evaluated against clinically assessed reference values. Model predictions were compared to clinical data using Pearson correlation coefficients (r), root mean square error (RMSE), and Bland-Altman analyses.

**Results:**

A strong agreement was observed between simulated and clinical data for tidal volumes (*r* = 0.925, *p* < 0.001, RMSE = 0.053 l) and driving pressures (*r* = 0.940, *p* < 0.001, RMSE = 1.328 mbar) across all PEEP levels. At initial PEEP level, a high image correlation (*r* > 0.8) was found between measured and simulated regional ventilation in five of seven patients where EIT data were available.

**Conclusions:**

This pilot study demonstrates the capability of the individualized, regionally resolved physics-based computational lung model to reproduce patient-specific respiratory mechanics and key features of regional ventilation patterns in ARDS. The strong agreement between simulated and measured data supports the potential of this approach to estimate the physiological response to ventilator adjustments and lays the groundwork for future studies integrating computational modeling into clinical decision-making.

**Trial registration:**

Registered at the German Clinical Trials Register (DRKS00017151, https://drks.de/search/en/trial/DRKS00017151) at 12th April 2019.

**Supplementary Information:**

The online version contains supplementary material available at https://doi.org/10.1186/s12931-026-03949-z.

## Background

Mechanical ventilation can be a life-saving treatment for critically ill patients with acute respiratory distress syndrome (ARDS) [1–3]. However, mechanical ventilation may itself damage the lungs through cyclic collapse and reopening of lung units (atelectrauma) or overdistension at high volumes (volutrauma and barotrauma). These mechanisms are referred to as ventilator-associated lung injury (VALI) (4–6]. In recent years, various lung-protective ventilation strategies have been investigated, of which only a few showed a clear positive effect on all-cause mortality [7, 8]. Other studies seem to improve outcomes for individual patients but do not have a significant overall effect [1, 9, 10].

A major obstacle for individualized protective ventilation is the limited clinical insight into lung behavior at a locoregional level. Because patients may respond very differently to the same ventilation strategies - ranging from benefit to potential harm - personalized assessment of lung pathophysiology, regional distensibility, and ventilation status could help identify those most likely to benefit. Especially when available in a predictive manner, such patient-specific insights could aid in identifying the best ventilatory strategies early in treatment.

Imaging techniques such as computed tomography (CT) and electrical impedance tomography (EIT) provide valuable insight into lung physiology and can support the personalization of ventilatory strategies [11–16]. CT images offer high spatial resolution, but only at the time of the examination. In contrast, EIT allows continuous monitoring of regional ventilation distribution at the bedside and is free from radiation exposure, but it provides only coarse spatial resolution. Interpreting EIT data accurately and correlating these with CT images findings to optimize ventilator settings remains a considerable challenge for physicians [17, 18]. Further, neither can provide predictive information on the patient’s response to different ventilatory strategies.

Some of these current methodological limitations may be overcome by advanced computational lung models. Such models have the potential to offer detailed, interpretable, and individual regional insight into the diseased lung under varying ventilation settings and may contribute significantly to the individualization of ventilatory management. In recent years, a physics-based computational lung model has been developed and extended that can be tailored to a patient’s lung geometry and respiratory mechanics: It realistically simulates tidal volume (V_T_), regional gas flow and tissue straining, recruitment/derecruitment and prevailing pressure in different areas of the lungs during spontaneous breathing and during the mechanical ventilation of diseased lungs [19–21]. This lung model has additionally been equipped with the capability to perform virtual EIT measurements, allowing validation of simulated regional ventilation distribution against clinical EIT recordings [22]. Prototypic digital twins of patients’ lungs developed using these modeling approaches were able to reproduce clinical measurements and showed strong potential to predict individual responses of mechanically ventilated patients to changes in ventilator settings [19–21].

In contrast to these earlier proof-of-concept applications in single-patient models, the aim of the present study was to evaluate the computational lung model in a prospective clinical pilot trial, testing its ability to predict patient-specific global and regional ventilation dynamics across systematically varied ventilatory conditions in a cohort of ventilated patients with ARDS.

## Methods

### Study design and setting

The monocentric prospective clinical pilot trial was conducted at the University Hospital Schleswig-Holstein in Kiel/Germany between 19 May 2021 and 2 March 2022. The study was registered at the German Clinical Trials Register (DRKS00017151, https://drks.de/search/en/trial/DRKS00017151) on 12 April 2019 and was approved by the local ethics committee (Ethics Committee of the Medical Faculty of the Kiel University, Germany, D485/17) before the beginning of patient recruitment. Written informed consent was obtained from the patients or their legal representatives prior to participation in the study. The study was set up according to the Equator Network STROBE guidelines [23]. All methods were performed following the relevant guidelines and regulations, as well as in accordance with the Declaration of Helsinki [24]. Patients were recruited and examined in the operative intensive care units. The data processing and model generation was performed at Institute for Computational Mechanics, Technical University of Munich, Germany.

### Participants

Patients were eligible for inclusion if they were 18 years or older and diagnosed with ARDS according to the Berlin definition [25]. An additional requirement was the availability of a thoracic CT scan that had been performed for clinical reasons within the preceding 24 h. Patients under the age of 18 years, pregnant women, patients with implanted electronic devices, burns or skin lesions in the thoracic region, severe chronic obstructive pulmonary disease, and patients with hemodynamic instability despite adequate fluid and catecholamine therapy were excluded.

### Experimental protocol

Patients were investigated during deep sedation with Propofol or Isoflurane, corresponding to a Richmond Agitation-Sedation Scale score of -5 [26], without spontaneous breathing activity. The ventilator (Elisa 800 VIT, Löwenstein Medical, Bad Ems, Germany) continuously measured and recorded airway pressure (P_aw_), air flow, esophageal pressure (P_eso_), inspiratory and expiratory concentrations of oxygen and carbon dioxide, and EIT data. For the recording of the EIT data, a 32-electrode belt (SensorBelt, SenTec, Landquart, Switzerland) was attached around the patient’s thorax in one slightly oblique plane in the area between the fourth and fifth intercostal space. Hemodynamic parameters, including heart rate, blood pressure, and central venous pressure were recorded using standard clinical monitoring equipment (MX750, Philips, Netherlands).

**Fig. 1:**
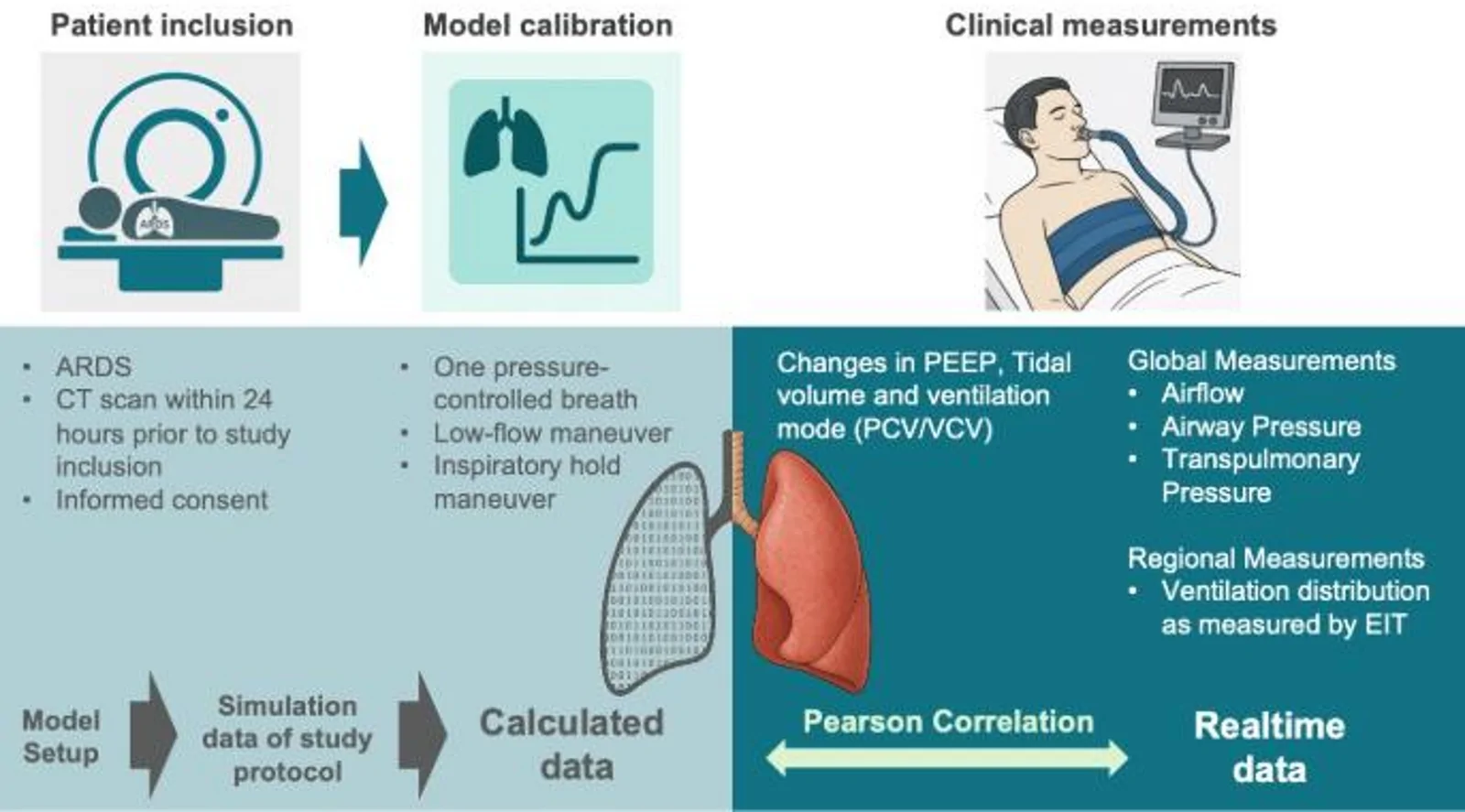
Sequence and key components of the study protocol

Correct positioning and functional coupling of the esophageal balloon catheter were validated using an occlusion test according to the method described by Baydur et al. [27]. Gentle manual thoracic compressions were applied. The catheter position was considered appropriate if the ratio of the resulting changes in esophageal pressure (ΔP_eso_) to airway pressure (ΔP_aw_) was between 0.8 and 1.2 during occlusion maneuver, indicating adequate transmission of pleural pressure changes to the esophageal balloon. Figure 1 gives an overview of the study protocol and the performed measurements.

**Fig. 2:**
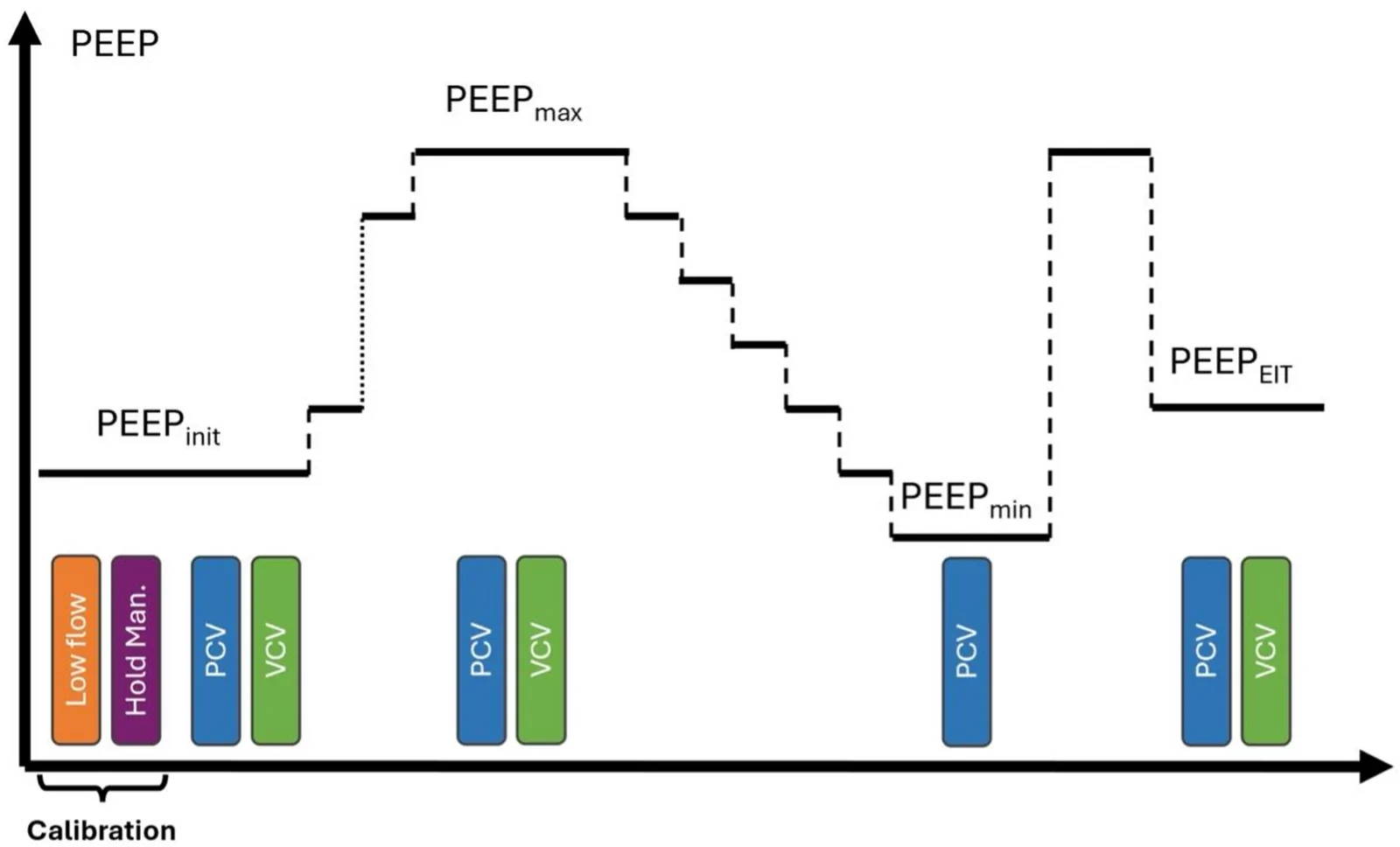
Sequence of PEEP levels and performed ventilation maneuvers

The study protocol started with the calibration beginning from a clinically set initial PEEP (PEEP_init_) – which was the same PEEP during CT scan – in pressure-controlled ventilation (PCV) mode with a constant low-flow maneuver (inspiration flow of 4 l/min) from PEEP_init_ up to a maximum airway pressure of 40 mbar or a maximum tidal volume (V_T_) of 12 ml/kg predicted body weight. Subsequently, another low-flow maneuver with the same inspiration flow was performed, starting at zero end-expiratory pressure up to a maximum V_T_ of 2000 ml or a maximum airway pressure of 40 mbar, whichever occurred first. In a following end-inspiratory hold maneuver, inspiration was prolonged for 5 s at constant end-inspiratory airway pressure. Next, during PCV at PEEP_init_ and the resulting V_T_ – both set for each patient individually according to the assessment of the physicians – the inspired fraction of oxygen FiO_2_ was increased once by 10% and set back again to allow calculation of end-expiratory lung volume (EELV) using the modified nitrogen dilution technique as described by Olegård et al. [28]. Ventilation mode was subsequently switched from PCV to volume-controlled ventilation (VCV) with identical V_T_ and no inspiratory pause and alternating inspiratory and expiratory hold maneuvers were applied before switching back to PCV. Afterward, PEEP was increased to a maximum PEEP of 24 mbar (PEEP_max_) in two to three steps, ensuring hemodynamic stability by adjusting vasopressor doses as needed, and held at this pressure level. An end-inspiratory airway occlusion of 5 s for assessment of airway plateau pressure (P_aw, plat_) and static respiratory system compliance (C_stat_) was performed. Equivalent to PEEP_init_, the ventilation mode was again temporarily switched to VCV to perform inspiratory and expiratory hold maneuvers. This was followed by a decremental PEEP trial, during which PEEP was reduced in 2 mbar steps from 24 mbar down to a minimum of 6 mbar (PEEP_min_). Each PEEP level was maintained for eight breath cycles, followed by an end-inspiratory and end-expiratory breath-hold maneuver to reassess P_aw, plat_ and C_stat_ before proceeding to the next PEEP step. After completion of the decremental PEEP trial and a short reset to PEEP_max_ to counteract alveolar collapse, PEEP was adjusted according to the crossover point of overdistension and alveolar collapse curves as detected by EIT, giving the EIT-derived crossover PEEP (PEEP_EIT_), according to the algorithm previously described [29, 30]. A schematic overview of all performed maneuvers is illustrated in Fig. 2.

The maximal (PEEP_max_ = 24mbar) and minimal (PEEP_min_ = 6mbar) PEEP levels are standardized for all patients, while the initial PEEP (PEEP_init_) and the PEEP as determined by EIT (PEEP_EIT_) are individually determined.

### Model setup

The model setup and validation pipeline consisted of two main components: the mechanical ventilation model and the EIT model. For the mechanical simulation model, we segmented the tracheal tree and lung lobes from the thoracic CT images and used a morphology-based tree-growing algorithm to create an anatomically accurate structure of the conducting airway tree [31]. The model’s non-linear pressure-volume characteristics - capable of replicating the sigmoidal behavior of lung tissue - were calibrated by an algorithm considering the patient’s respiratory mechanics during PCV for global parameters and the CT-derived pathology state for local parameters, including the integration of edematous fluid, pressure- and time-dependent recruitment/derecruitment dynamics, as well as a ventral-to-dorsal pressure gradient as introduced in previous work [21]. For this purpose, we considered a subset of the ventilation data at PEEP_init_, that did not overlap with the time intervals used for evaluation at PEEP_init_. This calibration data consisted of one low-flow maneuver, a single normal breath cycle, and the end-inspiratory hold maneuver. For this pilot study, the parameters of the mechanical model were manually optimized for each patient individually according to the procedure described in Geitner et al. [21]. The mechanical simulations were performed with the open-source multiphysics framework 4 C [32]. For the EIT model, we adapted the EIT-based regional validation method introduced by Roth et al. [22] and combined it with the anatomically informed reconstruction approach presented by Ludwig et al. [33]. The EIT model is based on a three-dimensional thorax model segmented from the patient CT images and uses the finite element method for the electrical simulations. An illustration of the mechanical ventilation model and the EIT reconstruction workflow, including the generation of a virtual EIT dataset, as well as a detailed description of both models, is provided in the Additional Files 1 and 2.

### Variables and evaluation metrics

To evaluate the predictive performance of the physics-based, patient-specific model, simulations were conducted using the ventilator settings (pressure, airflow, and volume profiles) defined by the study protocol. The resulting model outputs were then compared to corresponding clinical measurements using correlation analyses. An overview of study variables is summarized in Table 1. The following evaluations were designed for bidirectional validation of the model. During PCV, airway pressure was prescribed to predict the resulting airflow and tidal volume, whereas during VCV, the air flow profile was prescribed to predict the resulting airway pressure. Thus, both modes provided complementary information on the model’s predictive performance.

These comparisons were performed at PEEP_init_, PEEP_max_, PEEP_min_ and PEEP_EIT_. The primary objective of this study was to assess the accuracy of model-predicted V_T_ and driving pressure (ΔP_aw,_ defined as the difference between plateau pressure and PEEP) across the varying PEEP levels. For V_T_, the model was provided with the clinically recorded airway pressure curve during PCV to simulate airflow. Model-derived airflow was then integrated over time and compared with ventilator-measured V_T_. For ΔP_aw_, the model received the clinically applied V_T_ during VCV to simulate airway pressures, and the resulting simulated ΔP_aw_ at the airway opening was compared with clinical values.

Secondary objectives included evaluating the model’s performance in predicting end-expiratory transpulmonary pressure (P_tp, ee_) and EELV. Although both are end-expiratory parameters, their prediction required the full airway pressure curve during PCV, as time-dependent recruitment and pressure equilibration are integral to the model. Airway pressure during PCV was prescribed as input, yielding simulated airflow and lung volume. From this, the esophageal pressure at end-expiration (P_eso, ee_) emerged as a model output, based on simulated lung volume and pleural pressure dynamics, with calibration performed only at baseline to match the measured P_eso_ at the catheter position P_tp, ee_ was then obtained as the difference between the prescribed PEEP and the model-predicted P_eso, ee_. The EELV was calculated based on the reconstructed lung geometry and model dynamics. The model-derived EELV (EELV_model_) was compared to the reference values for EELV calculated using the modified nitrogen dilution technique (EELV_breathgas_) [28]. For additional comparison, EELV was also calculated directly from Hounsfield units of the CT scan (EELV_CT_). As both EELV_model_ and EELV_CT_ are derived from the same thoracic CT dataset, their comparison does not constitute an independent validation. Instead, it serves as an internal consistency check, verifying that the modeling pipeline faithfully translates the CT-derived geometry into an end-expiratory volume and does not drift to a different baseline level due to time-dependent effects. Accordingly, only the comparison against EELV_breathgas_, obtained by the methodologically independent modified nitrogen dilution technique, is regarded as a genuine validation of absolute volume prediction.

Further objectives included assessing the spatial agreement between predicted and measured tidal EIT images averaged over 12 PCV breaths. Regional ventilation was predicted from both PCV and VCV inputs, reflecting the availability of clinical EIT data in both modes. Model predictions were compared with clinical EIT data using two complementary approaches: [1] pixel-wise correlation between predicted and measured EIT images, and [2] comparison of established clinical indices, including regional fraction of ventilation (F_dorsal_, F_left_) and the centers of ventilation (CoV_rl_ and CoV_vd_). The fractions F_dorsal_ and F_left_ were calculated for the dorsal and left thoracic halves, respectively, while CoV_rl_ and CoV_vd_ represented the centers of ventilation along the right–left and ventral–dorsal axes. An illustrative figure defining the clinical indices is provided in the Additional File 3.

**Table 1:** Overview of study variables, model inputs and outputs, and evaluation methods

Parameter/ Model-Output	Model-Input	Description of Model Prediction
Tidal Volume (V_T_)	Airway pressure curve (PCV) as prescribed by ventilator	Integrated simulated airflow
Driving Pressure (ΔP_aw_)	Volume Flow curve (VCV) as prescribed by the ventilator	Simulated tidal pressure difference during VCV
End-expiratory Transpulmonary Pressure (P_tp, ee_)	Airway pressure curve (PCV) as prescribed by ventilator	Simulated difference of PEEP and end-expiratory esophageal pressure
End-Expiratory Lung Volume (EELV)	Airway pressure curve (PCV) as prescribed by ventilator	Simulated EELV after several cycles at same PEEP level
Regional Ventilation (EIT)	Airway pressure curve (PCV) or Volume Flow Curve (VCV), as prescribed by ventilator	Simulated EIT images based on regional ventilation as predicted by the model

Additional model insights included the end-expiratory fraction of open lung volume at different PEEP levels, derived from the overall percentage of recruited model volume, and the lung’s edema volume V_edema_ and tissue volume V_tissue_. The latter parameters were estimated during the model calibration as described by Geitner et al. and are constant throughout the simulated ventilation [21]. Their plausibility was supported by the model’s ability to reproduce the measured pressure-volume behavior. As there is no clinical reference available for these additional model insights, we present the respective results in the Additional File 4.

### Statistical methods

All statistical analyses were performed using Python (version 3.9) with the SciPy library (version 1.10). The normality of the residuals between measurements and simulation was assessed using the Shapiro-Wilk test. Method comparisons were done by correlation scatter plots, Bland-Altman plots, and analyses of the RMSE. The Pearson correlation coefficient r was calculated to assess the strength of the relationship between the clinically measured and model-predicted quantities. A p-value < 0.05 was considered statistically significant and we did not adjust for multiple comparisons. Given the small patient cohort, we report correlations and Bland-Altman limits of agreement pooled across PEEP levels as condensed descriptive summaries. These pooled quantities are exploratory and descriptive: they do not account for within-patient clustering and may overestimate the strength of the association. We therefore additionally report the RMSE per evaluation condition (Tables 2 and 4) and per patient (Additional File 8).

A power-based sample size calculation was not performed. To account for anatomical differences and various distribution patterns of ventilation disorders, a sample size of 10 patients was deemed appropriate for this pilot study.

## Results

### Analyzed data

A total of 10 patients were included. Owing to the exploratory design, no formal screening log was maintained. Data were analyzed for both primary and secondary outcome measures across different PEEP levels (PEEP_init_, PEEP_max_, PEEP_min_, and PEEP_EIT_).

For the primary objectives, a total of 37 measurements of V_T_ were available (PEEP_init_: *n* = 10; PEEP_max_: *n* = 9; PEEP_min_: *n* = 9; PEEP_EIT_: *n* = 9). Driving pressure was assessed in 18 measurements (PEEP_init_: *n* = 9 and PEEP_EIT_: *n* = 9). For the secondary objectives, transpulmonary pressures were analyzed in 33 measurements (PEEP_init_: *n* = 9; PEEP_max_: *n* = 8; PEEP_min_: *n* = 8; PEEP_EIT_: *n* = 8). EELV was available for all 10 patients. EIT-derived data were available in 25 PCV measurements (PEEP_init_: *n* = 7; PEEP_max_: *n* = 6; PEEP_min_: *n* = 6; PEEP_EIT_: *n* = 6) and 12 VCV measurements (PEEP_init_: *n* = 6; PEEP_EIT_: *n* = 6), i.e. 37 EIT datasets in total. Data availability and data exclusion are reported in Additional File 5.

### Patient baseline data

The age range of the patients (7 male/3 female, height 177 ± 10 cm, weight 88 ± 17 kg, Body-Mass-Index 27.8 ± 4.8 kg/m^2^) was 25 to 81 years with a mean of 61 ± 16 years. Four patients had mild ARDS, four patients presented with moderate ARDS, and two patients fulfilled the criteria for severe ARDS. P_a_O_2_/FiO_2_ at baseline was 174 ± 68 mmHg. Median PEEP_init_ level was 13.5 [10-16.5] mbar, V_T_ was 549 ± 134 ml and driving pressure was 15.6 ± 3.6 mbar. A detailed tabular overview of patient characteristics is presented in Additional File 6.

### Primary and secondary objectives

**Fig. 3:**
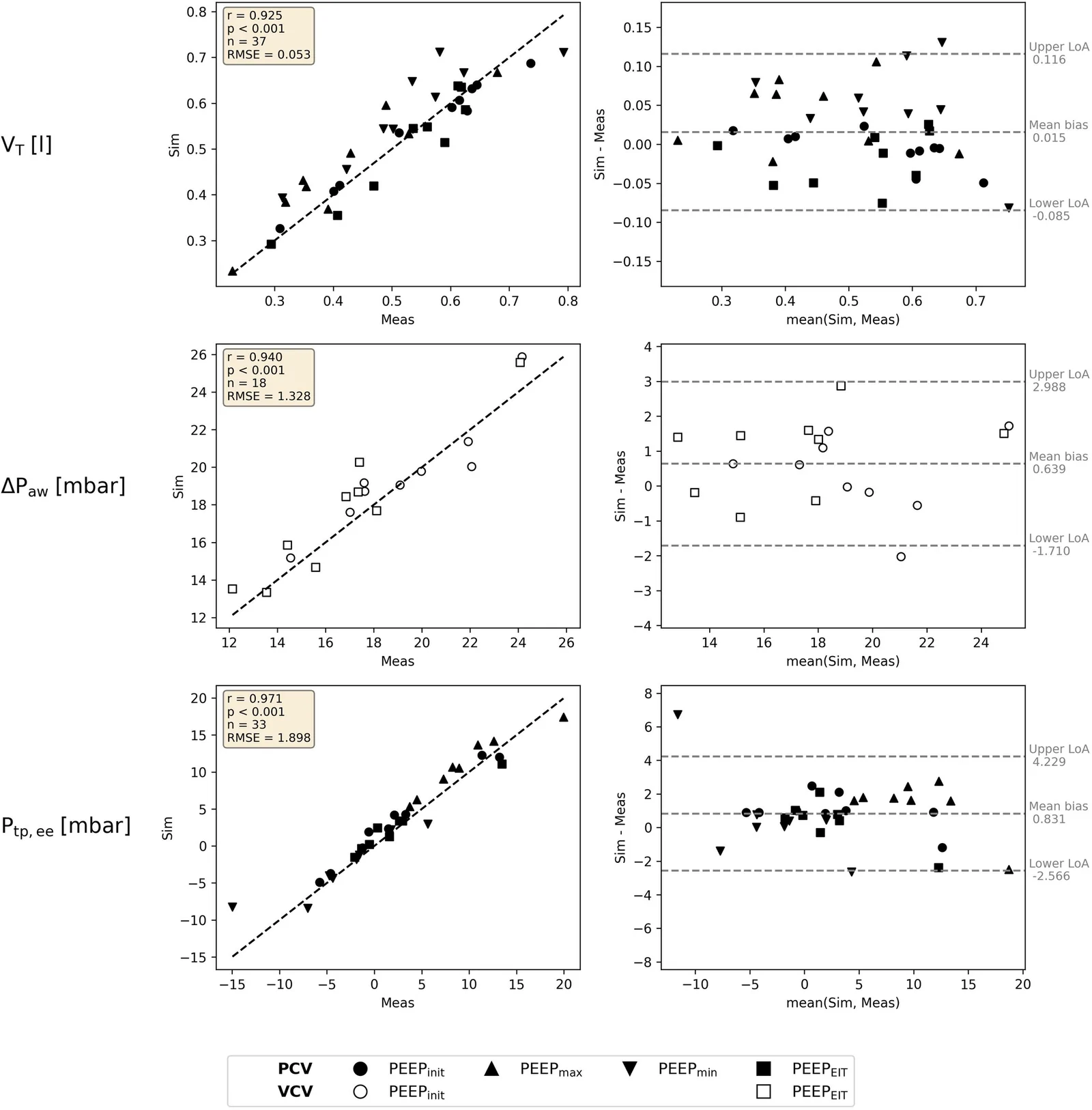
Correlation and Bland-Altman plots for V_T_, ΔP_aw_, and P_tp, ee_ between model outputs and real time measurements

Regarding our primary objectives, we observed a strong correlation between simulated and clinical V_T_ (*r* = 0.925, *p* < 0.001, RMSE = 0.053 l) as well as ΔP_aw_ (*r* = 0.940, *p* < 0.001, RMSE = 1.328 mbar) across all considered PEEP levels. Visualized individual comparisons of measured and simulated airway pressure and tidal volume curves at PEEP_init_ and PEEP_EIT_ are provided in the Additional File 7. For the secondary objective P_tp, ee_, we also observed a strong correlation between simulated and clinical P_tp, ee_ (*r* = 0.971, *p* < 0.001, RMSE = 1.898 mbar) across all considered PEEP levels. Figure 3 shows the correlation and the Bland-Altman diagrams of the individual outcome measures. As these pooled coefficients do not account for repeated measurements within patients, Table 2 further reports the correlation and RMSE on an evaluation-condition level showing the agreement for V_T_, ΔP_aw_, and P_tp, ee_, at the respective PEEP levels, and Additional File 8 reports the corresponding patient-level RMSE.

**Table 2:** Model performance for global mechanical parameters stratified by PEEP level and ventilator mode. At PEEP_init_, the calibration condition, the comparison quantifies agreement rather than predict an unseen ventilatory setting; PEEP_max_, PEEP_min_ and PEEP_EIT_ are out-of-calibration conditions

Parameter [unit]	Mode	PEEP level (*n*=#cases)	RMSE (condition)	*r* & *p* (condition)	LoA (condition)	RMSE (pooled)	*r* & *p* (pooled)	LoA (pooled)
V_T_ [l]	PCV	init (*n* = 10)	0.024	*r* = 0.991; *p* < 0.001	[-0.054, 0.041] (bias=-0.007)	0.053	*r* = 0.925; *p* < 0.001	[-0.085, 0.116] (bias = 0.015)
	PCV	max (*n* = 9)	0.059	*r* = 0.939; *p* < 0.001	[-0.051, 0.130] (bias = 0.040)			
	PCV	min (*n* = 9)	0.077	*r* = 0.893; *p* = 0.001	[-0.067, 0.170] (bias = 0.051)			
	PCV	EIT (*n* = 9)	0.039	*r* = 0.958; *p* < 0.001	[-0.089, 0.050] (bias=-0.020)			
ΔP_aw_ [mbar]	VCV	init (*n* = 9)	1.147	*r* = 0.922; *p* < 0.001	[-1.975, 2.607] (bias = 0.316)	1.328	*r* = 0.940; *p* < 0.001	[-1.710, 2.988] (bias = 0.639)
	VCV	EIT (*n* = 9)	1.488	*r* = 0.952; *p* < 0.001	[-1.399, 3.323] (bias = 0.962)			
P_tp, ee_ [mbar]	PCV	init (*n* = 9)	1.382	*r* = 0.990; *p* < 0.001	[-0.993, 2.987] (bias = 0.997)	1.898	*r* = 0.971; *p* < 0.001	[-2.566, 4.229] (bias = 0.831)
	PCV	max (*n* = 8)	2.056	*r* = 0.964; *p* < 0.001	[-1.807, 4.572] (bias = 1.383)			
	PCV	min (*n* = 8)	2.623	*r* = 0.923; *p* = 0.001	[-4.825, 5.922] (bias = 0.549)			
	PCV	EIT (*n* = 8)	1.269	*r* = 0.984; *p* < 0.001	[-2.165, 2.916] (bias = 0.376)			

The results regarding the comparison end-expiratory lung volume of EELV_model_ to the EELV_breathgas_ and EELV_CT_ were heterogeneous: While EELV_model_ resembled the reference value EELV_CT_ for all patients, EELV_breathgas_ of Patient 4, Patient 7, and Patient 10 were different from EELV_model_ and EELV_CT_. For patients 6 and 9, EELV_breathgas_ and EELV_model_ were relatively close. For technical reasons, EELV_breathgas_ was unavailable for patients 1–3 and 5. Table 3 summarizes the EELV results.

**Table 3:** Endexpiratory lung volume (EELV) values in individual patients

Patient	1	2	3	4	5	6	7	8	9	10
EELV_breathgas_ (l)	–	–	–	3.21	–	1.70	3.50	1.95	1.99	3.58
EELV_CT_ (l)	1.11	3.30	4.30	1.55	3.17	1.70	1.96	2.27	1.96	1.91
EELV_model_ (l)	1.07	3.27	4.29	1.50	3.20	1.67	1.98	2.29	1.96	1.87

**Fig. 4:**
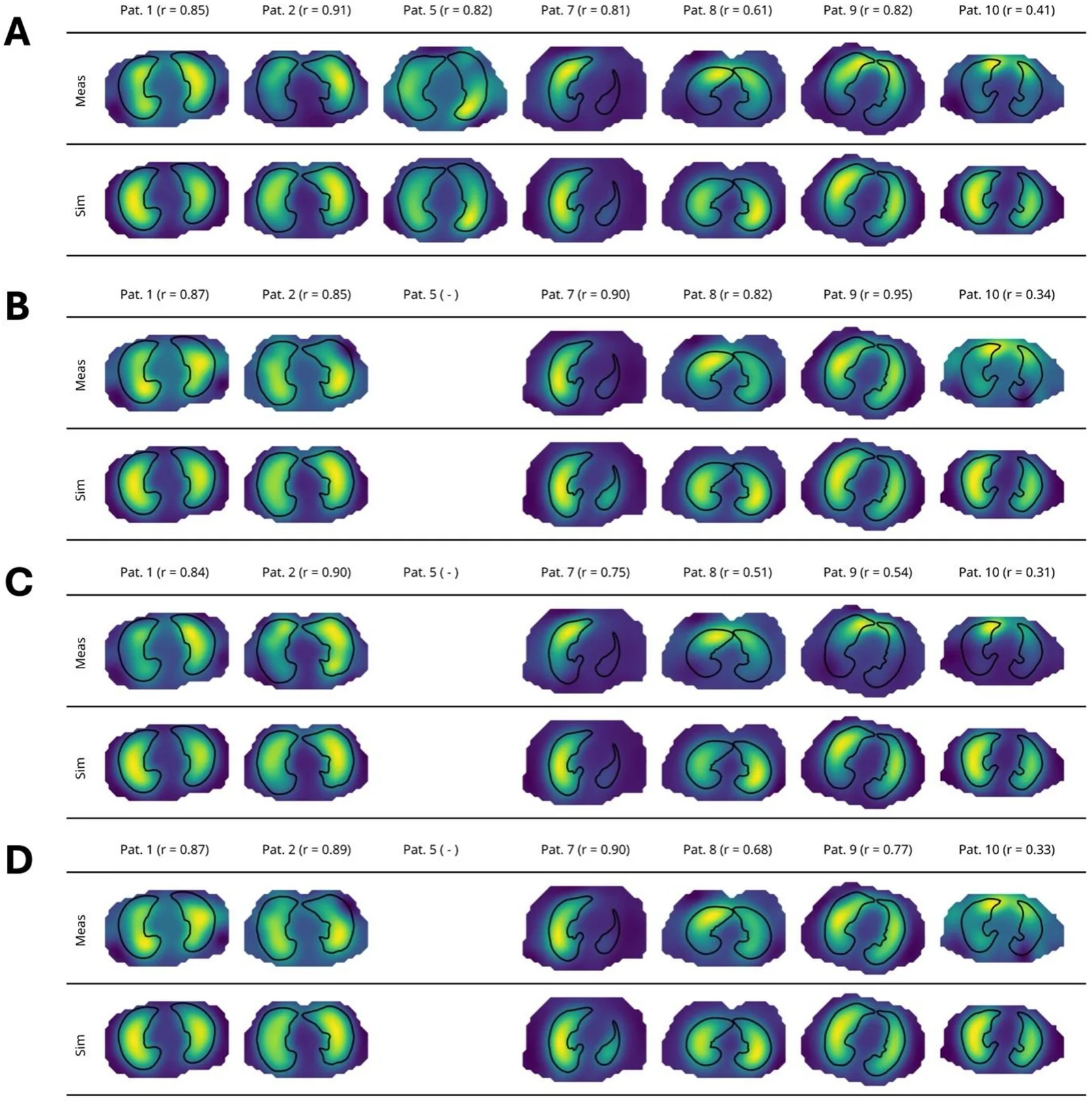
EIT images for clinical and simulated EIT measurements

A comparative analysis of EIT images obtained from clinical measurements and the simulated EIT are presented in Fig. 4. The images show the tidal ventilation distribution in PCV mode at PEEP_init_, PEEP_min_, PEEP_max_ and PEEP_EIT_. To evaluate the consistency between measured and simulated data, we computed the pixel-wise Pearson correlation for the paired images. At the initial PEEP level, we observed high to very high correlation for five patients (*r* > 0.8 for Patient 1, 2, 5, 7 and 9). We obtained less correlation for Pat. 8 and Pat. 10 (*r* = 0.61 and *r* = 0.41, respectively). This observation also aligns with the visual comparison, where the left–right and dorsal–ventral distribution of ventilation in these patients have a stronger mismatch.

Pairwise schematic EIT images in A for PEEP_init_, B for PEEP_max_, C for PEEP_min_ and D for PEEP_EIT_. A high tidal ventilation is indicated by yellow, and low ventilation by violet.

**Fig. 5:**
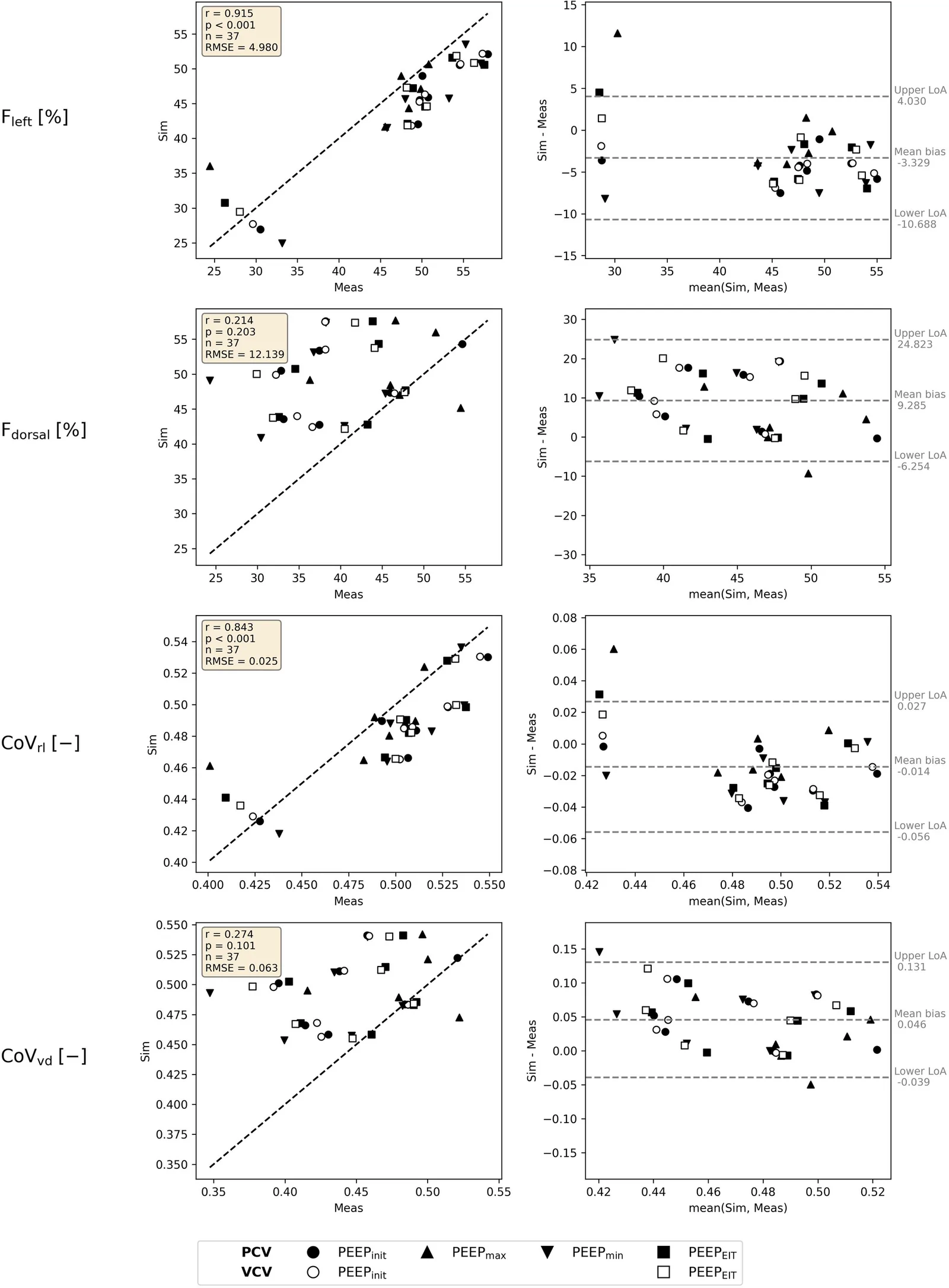
Correlation and BlandAltman analysis of EIT-based regional ventilation metrics

Figure 5 demonstrates the agreement of model-predicted and measured EIT by means of the common clinical measures F and CoV.

For the EIT quantities, we observed higher Pearson correlations for measures quantifying right-left (rl) distribution (F_left_: *r* = 0.915, *p* < 0.001, RMSE = 4.980% and CoV_rl_: *r* = 0.843, *p* < 0.001, RMSE = 0.025) than for the measures quantifying ventral-dorsal (vd) distribution (F_dorsal_: *r* = 0.214, *p* = 0.203, RMSE = 12.139% and CoV_vd_: *r* = 0.274, *p* = 0.101, RMSE = 0.063). Table 4 reports these results on an evaluation condition level allowing for a more detailed analysis of the model performance for the different PEEP levels and ventilator modes. In this table, the dependency of the ventro-dorsal mismatch in F_dorsal_ and CoV_vd_ on the PEEP level can be observed and we can state that this mismatch is significantly smaller at PEEP_max_ compared to the other PEEP levels. The interested reader is referred to Additional File 9 where we analyze this most pronounced mismatch in more detail.

**Table 4:** Condition-level model performance for regional ventilation parameters (F_left_, F_dorsal_, CoV_rl_, CoV_vd_) stratified by PEEP level and ventilator mode

Parameter [unit]	Mode	PEEP level (*n*=#cases)	RMSE (condition)	*r* & *p* (condition)	LoA (condition)	RMSE (pooled)	*r* & *p* (pooled)	LoA (pooled)
F_left_ [%]	PCV	init (*n* = 7)	4.800	*r* = 0.974; *p* < 0.001	[-8.325, -0.543] (bias=-4.434)	4.980	*r* = 0.915; *p* < 0.001	[-10.688, 4.030] (bias=-3.329)
	PCV	max (*n* = 6)	5.411	*r* = 0.870; *p* = 0.024	[-11.218, 11.963] (bias = 0.373)			
	PCV	min (*n* = 6)	5.642	*r* = 0.969; *p* = 0.001	[-10.367, 0.218] (bias=-5.075)			
	PCV	EIT (*n* = 6)	4.954	*r* = 0.957; *p* = 0.003	[-11.451, 5.409] (bias = 3.021)			
	VCV	init (*n* = 6)	4.634	*r* = 0.990; *p* < 0.001	[-7.600, 1.171] (bias = 4.386)			
	VCV	EIT (*n* = 6)	4.348	*r* = 0.961; *p* = 0.002	[-9.458, 2.965] (bias=-3.246)			
F_dorsal_ [%]	PCV	init (*n* = 7)	12.416	*r* = 0.321; *p* = 0.483	[-5.730, 25.653] (bias = 9.962)	12.139	*r* = 0.214; *p* = 0.203	[-6.254, 24.823] (bias = 9.285)
	PCV	max (*n* = 6)	8.185	*r*=-0.015; *p* = 0.977	[-12.172, 19.383] (bias = 3.605)			
	PCV	min (*n* = 6)	15.089	*r* = 0.089; *p* = 0.867	[-5.828, 30.740] (bias = 12.456)			
	PCV	EIT (*n* = 6)	10.606	*r* = 0.295; *p* = 0.571	[-5.470, 22.287] (bias = 8.409)			
	VCV	init (*n* = 6)	13.176	*r* = 0.055; *p* = 0.917	[-2.941, 25.674] (bias = 11.366)			
	VCV	EIT (*n* = 6)	12.168	*r* = 0.250; *p* = 0.632	[-5.697, 25.291] (bias = 9.797)			
CoV_rl_ [–]	PCV	init (*n* = 7)	0.024	*r* = 0.933; *p* = 0.002	[-0.048, 0.008] (bias=-0.020)	0.025	*r* = 0.843; *p* < 0.001	[-0.056, 0.027] (bias=-0.014)
	PCV	max (*n* = 6)	0.028	*r* = 0.697; *p* = 0.124	[-0.057, 0.063] (bias = 0.003)			
	PCV	min (*n* = 6)	0.026	*r* = 0.917; *p* = 0.010	[-0.053, 0.009] (bias=-0.022)			
	PCV	EIT (*n* = 6)	0.026	*r* = 0.859; *p* = 0.029	[-0.062, 0.037] (bias=-0.012)			
	VCV	init (*n* = 6)	0.024	*r* = 0.948; *p* = 0.004	[-0.048, 0.009] (bias=-0.020)			
	VCV	EIT (*n* = 6)	0.024	*r* = 0.886; *p* = 0.019	[-0.055, 0.025] (bias=-0.015)			
CoV_vd_ [–]	PCV	init (*n* = 7)	0.062	*r* = 0.407; *p* = 0.364	[-0.031, 0.130] (bias = 0.049)	0.063	*r* = 0.274; *p* = 0.101	[-0.039, 0.131] (bias = 0.046)
	PCV	max (*n* = 6)	0.044	*r* = 0.019; *p* = 0.972	[-0.070, 0.103] (bias = 0.017)			
	PCV	min (*n* = 6)	0.078	*r* = 0.176; *p* = 0.739	[-0.043, 0.166] (bias = 0.061)			
	PCV	EIT (*n* = 6)	0.056	*r* = 0.315; *p* = 0.543	[-0.037, 0.121] (bias = 0.042)			
	VCV	init (*n* = 6)	0.066	*r* = 0.242; *p* = 0.644	[-0.021, 0.132] (bias = 0.055)			
	VCV	EIT (*n* = 6)	0.064	*r* = 0.279; *p* = 0.592	[-0.040, 0.139] (bias = 0.049)			

## Discussion

In this prospective pilot study, we demonstrated that a patient-specific, physics-based lung model can reproduce global and regional ventilation patterns in patients with respiratory failure with high accuracy. Using CT data and minimal clinical input for generating an individualized physics-based, patient-specific model, the model predictions showed strong agreement with measured V_T_, ΔP_aw_ and P_tp, ee_, underlining the validity of this approach despite the heterogeneity of ARDS.

Agreement in global evaluation parameters was strongest near the calibration point at PEEP_init_ and at PEEP_EIT_, while moderate deviations occurred at PEEP_min_ and PEEP_max_. This result is not unexpected, given that the model calibration relied exclusively on data obtained at the initial PEEP level. Note that calibration and evaluation at PEEP_init_ were performed on non-overlapping time intervals, recorded several minutes apart, meaning that we did not evaluate the model performance with the same waveforms that we used for calibration. The evaluation at PEEP_init_ therefore also reflects any change in patient condition or ventilatory state occurring between the two recordings, rather than solely reproducing the calibration data. Despite being calibrated from a single PEEP setting covering a tidal pressure range of ~ 10-30mbar, the model retains good extrapolation potential at the more extreme pressure levels PEEP_min_ and PEEP_max_. Note, for the region around PEEP_init_, we cannot exclude effects of overfitting which may contribute to an overestimation of predictive performance in this pressure range. Importantly, we did not see a systematic deterioration of predictions at PEEP_EIT_, indicating that the temporal distance between the calibration maneuvers and later ventilation steps did not significantly degrade the model’s performance. Furthermore, we can observe clear effects of recruitment-derecruitment during the study period for some of the patients (opening/closing of ~ 5% lung volume from PEEP_init_ to PEEP_EIT_. See Additional File 4). In addition, the model showed good agreement with transpulmonary pressure, indicating its ability to capture esophageal pressure dynamics. Occasional outliers, likely caused by artifacts such as swallowing or small shifts in esophageal probe placement, did not affect the overall correlation but highlight the need for robust procedures in measuring and interpreting esophageal pressure. Our findings are consistent with results reported by other research groups using patient-specific virtual lung models. In line with the work of Zhou et al., who demonstrated high predictive accuracy for PEEP-induced changes in airway pressures and recruited lung volume up to 12 cmH₂O (R² up to 0.90) [34], and Caljé-van der Klei et al., who showed robust prediction of elastance and distension mechanics across ventilation modes (R² up to 0.95) [35], our data further support the reliability of physiologically informed digital twin models to anticipate individual lung responses to PEEP adjustments with small absolute prediction errors.

The clinical implications of the observed RMSE values warrant careful consideration. For V_T_, the condition-level RMSE ranged from 24 to 77 ml, which must be interpreted in the context of current lung-protective ventilation recommendations of less than 6 ml/kg predicted body weight. In a 70 kg patient, this threshold corresponds to a target V_T_ of approximately 420 ml, meaning that a model error of up to 77 ml could potentially lead to a clinically relevant overestimation or underestimation of the delivered tidal volume. This underscores the importance of not applying the model predictions in isolation at its current stage of development, but rather as a complementary tool alongside direct clinical monitoring. Future refinements of the model, including an improved formulation of the pleural pressure boundary condition and an automatized parameter calibration approach may further reduce prediction errors and ultimately support a more autonomous clinical application.

For ΔP_aw_, the pooled RMSE of 1.3 mbar is considered clinically tolerable, particularly given that current recommendations suggest keeping driving pressure below 15 mbar. An error of this magnitude is unlikely to misclassify a patient as safe or at risk, provided that predictions are interpreted with an appropriate safety margin.

Similarly, the RMSE of approximately 1.9 mbar for P_tp, ee_ appears acceptable in clinical practice, where esophageal pressure-guided ventilation serves as one of several decision-making parameters rather than the sole determinant of ventilator settings. Taken together, these findings suggest that while the model demonstrates promising predictive accuracy, its current precision may be best suited to guiding ventilator adjustments in a supportive capacity, with final clinical decisions remaining with the treating physician.

Our assessment of EELV revealed good agreement between EELV_model_ and EELV_CT_ for most patients. However, this agreement reflects the internal consistency of the modeling pipeline rather than independent predictive accuracy, since both quantities originate from the same CT dataset. The only methodologically independent reference, EELV_breathgas_, showed substantial discrepancies in several patients when compared to both CT and model predictions. These discrepancies may arise from several sources: timing differences between CT acquisition and the bedside EELV measurement, measurement noise, and variations in the underlying clinical condition, may cause these discrepancies between the actual patient EELV and the model-derived volume. When data were complete and artifact-free (e.g., in Patients 6 and 9), the EELV_breathgas_ more closely resembled the model and CT values. It should further be noted that the EELV measurement method by Olegård et al. assumes stable ventilation and homogeneous lung function and provides only global rather than regional information which makes it error-prone in critically ill patients [36]. As a functional measure it reflects ventilated rather than aerated volume, so part of the discrepancy is methodological rather than measurement error. Given these constraints on both the reference method and the model, our EELV results should be regarded as exploratory. Model-based EELV estimation may nevertheless be clinically relevant, not as a replacement of reference methods per se, but as a complementary approach whose predictive accuracy warrants confirmation in future studies with independent reference measurements.

Comparing the spatial ventilation distribution between clinical EIT images and model predictions in Fig. 4, we found a very good (*r* > 0.8) pixel-wise correlation in five of seven patients at PEEP_init_, reflected visually by a close match in the right-left and the dorsal–ventral distribution of tidal ventilation. It should be noted that three patients were excluded from this analysis due to technical EIT recording failures; two of these patients had a BMI above the cohort mean, suggesting that they may have presented greater challenges both for EIT belt placement and for dorso-ventral model predictions. For the patients 8 and 10 the pixel-wise correlations were significantly weaker at PEEP_init_. At the other PEEP levels the results of the regional comparison appear heterogeneous across the patients with weaker correlations at PEEP_min_ and stronger correlations at PEEP_max_. The regional ventilation metrics shown in Fig. 5; Table 4 highlight that there is a clear difference in prediction quality between the left–right and the dorsal–ventral axis with a higher mismatch on the dorsal–ventral axis. The model tended to overestimate dorsal ventilation. In Additional File 9 we show additional contextual data analyzing the mismatch in prediction quality in dorsal-ventral direction indicating an association of the mismatch with the PEEP level. We observed the smallest mismatch at PEEP_max_. We hypothesize that neglecting the dorsally-strain resisting influence of the abdomen and the abdominal organs on the external pressure acting on the model tissue elements is likely contributing to this mismatch. So far, only the global volume-dependent pleural pressure and the pressure gradient across the lung due to the organ’s own weight are considered in the lung model. Nevertheless, the consistent agreement observed in several patients underscores that the lung model can reproduce key features of regional ventilation distribution. Future refinements addressing pressure gradients related to lung fluid accumulation, alveolar fluid content in the EIT model, and patient positioning may further reduce dorsal–ventral discrepancies. Beyond these technical limitations, recent clinical studies suggest that EIT use in patients with ARDS may be associated with beneficial effects (37–39], highlighting the clinical relevance of personalization in ventilatory treatment that may be improved by simulation-based approaches. From a clinical perspective, the observed prediction error for F_dorsal_ of approximately 12% warrants careful consideration. In ARDS patients, dorsal lung regions are particularly prone to atelectasis and consolidation, and accurate prediction of regional ventilation distribution is essential for optimizing PEEP settings and minimizing the risk of ventilator-induced lung injury. An overestimation of dorsal aeration by the model could therefore lead to suboptimal ventilator adjustments, underscoring the need for improved modeling of gravitational effects and the influence of abdominal structures on regional lung mechanics before bedside adoption can be considered.

Additional model-derived insights without a clinical reference included the fraction of open lung volume at each PEEP level and a model-derived estimate of edema volume. We report these exploratory endpoints in Additional File 4. Although not independently validated in this study, these outputs illustrate a key strength of physics-based modeling: the ability to provide mechanistic, patient-specific quantities that are difficult or impossible to measure directly at the bedside.

Several approaches to lung modeling in ARDS have been proposed, each with specific strengths and limitations. Compartment models are computationally efficient but lack spatial resolution and cannot capture regional heterogeneity or recruitment/derecruitment dynamics [40]. Data-driven methods, including machine learning, can detect nonlinear relationships but require large datasets, often lack generalizability and individualizability, and provide little physiological interpretability, limiting clinical acceptance [41]. Imaging techniques such as CT or EIT offer spatial insights, but CT is unsuitable for bedside use and EIT suffers from low spatial resolution and susceptibility to artefacts [12]. Recently, Dunphy et al. [42] inferred regional elastance and recruitability from computed tomography acquired at two airway pressures, which avoids calibration from ventilator waveforms but requires an additional CT acquisition. Importantly, in contrast to our previous work by Rixner et al. [43], which was based on a subset of the present clinical data and did not account for recruitment and derecruitment, the current study applies an extended computational model incorporating these mechanisms. By integrating CT-derived morphology with mechanistic simulation, this patient-specific framework enables predictive and physiologically interpretable assessment of global and regional ventilation.

Such individualized models may serve as a foundation for physiologically informed, personalized ventilatory management at the bedside. A key clinical advantage lies in estimating parameters otherwise difficult to obtain, such as transpulmonary pressure, without invasive monitoring. As esophageal manometry is costly, requires expertise, and is rarely used outside specialized centers [44, 45], a validated noninvasive alternative could reduce resource use, avoid unnecessary catheterization, and improve patient comfort. Beyond parameter estimation, patient-specific modeling may facilitate individualized ARDS treatment strategies. Approaches tailored to underlying pathophysiological phenotypes of ARDS have been associated with improved outcomes, and recent literature highlights the need to further refine and validate such strategies across diverse populations [1, 46, 47]. In this context, individualized modeling may also identify patients in whom deviations from guideline-based recommendations are physiologically justified. Our modeling framework contributes to this effort by providing a physics-based, patient-specific approach to support more nuanced, pathophysiology-guided ventilatory decision-making.

Several limitations warrant consideration. First, the study cohort was small with heterogeneous ARDS presentations, and our results thus need confirmation in larger, more standardized populations. The relatively small sample size limits the statistical power of the correlation analyses, and correlation coefficients should therefore be interpreted with caution, as they may overestimate model performance in small samples. In addition, each patient contributed several measurements across PEEP levels to the analyses in Figs. 3 and 5, so the correlations are pooled across non-independent observations and may overestimate agreement. We therefore base our interpretation of predictive accuracy on the condition-level and patient-level RMSE values, which are not affected by this pooling. Due to the small number of independent patients, we did not apply mixed-effects modeling. At the same time, the cohort closely mirrors the complexity encountered in clinical practice — patient inclusion was governed by the stringent requirements of the study protocol, including ARDS diagnosis, CT availability within 24 h, successful esophageal catheter placement, and informed consent, reflecting real-world clinical constraints rather than deliberate selection. Importantly, the cohort included patients with suboptimal measurement conditions and highly heterogeneous CT morphology, and the fact that the model yielded consistent and promising results under these conditions supports its robustness and potential for real-world applicability.

Second, although most patients underwent the full set of ventilatory maneuvers, missing data points and irregularities in esophageal pressure and airflow measurements reflect the inherent difficulties of conducting clinical research in critically ill patients. Third, the current model calibration relies heavily on quasi-static pressure-volume data obtained near the initial PEEP level and we may tend to overfit the curve there; predictions at substantially different pressure levels might be improved by including additional data points covering a broader pressure range, albeit at the cost of more elaborate clinical maneuvers. Fourth, our EIT model approximates thoracic geometry and tissue resistivity based on a single CT dataset in the supine position, without accounting for the movement of the chest wall and the lungs, or a more refined resolution of the electrical tissue resistivity, or fluid distribution over time. Finally, while computational speed was sufficient for retrospective analyses, real-time application in the ICU will require further optimization and automation of our current modeling and calibration pipeline. A novel implementation of the computational model is currently in development and has already achieved substantial speedups. Moreover, a semi-automatic modeling pipeline has recently been developed that markedly accelerates model generation, thereby facilitating its potential integration into time-critical ICU workflows [43].

In summary, our personalized, physics-based computational lung model demonstrated promising predictive capabilities and accuracy in replicating global mechanical parameters and regional ventilation patterns in mechanically ventilated ARDS patients. Despite the inherent challenges of bedside measurements, especially in critically ill populations, the close agreement between simulations and measurements underscores the potential clinical value of physics-based, patient-specific models. The present study is a retrospective, offline analysis. Enhanced modeling of fluid dynamics, time-varying thoracic mechanics, and automated calibration workflows are expected to enable near real-time predictions of individual patients’ responses to different ventilatory strategies in future. The present results, including strong global agreement across PEEP levels and reliable right-to-left as well as exploratory dorsal-to-ventral regional predictions, provide a concrete basis for prospective studies testing whether such model-guided PEEP titration translates into measurable clinical benefit.

## Supplementary Information

Below is the link to the electronic supplementary material.

Supplementary Material 1

Supplementary Material 2

Supplementary Material 3

Supplementary Material 4

Supplementary Material 5

Supplementary Material 6

Supplementary Material 7

Supplementary Material 8

Supplementary Material 9

## Data Availability

The data that support the findings of this study are available on request from the corresponding author. The data are not publicly available due to privacy or ethical restrictions.

## Abbreviations

ARDS: Acute respiratory distress syndrome
VALI: Ventilator-associated lung injury
CT: Computed tomography
EIT: Electrical impedance tomography
PEEP: Positive end-expiratory pressure
PCV: Pressure-controlled ventilation
VCV: Volume-controlled ventilation
FiO_2_: Inspired fraction of oxygen
P_a_O_2_/FiO_2_: Ratio of arterial oxygen partial pressure to inspired fraction of oxygen
PEEP_init_: Initial clinically set PEEP
PEEP_max_: PEEP at 24 mbar
PEEP_min_: PEEP at 6 mbar
PEEP_EIT_: PEEP set after determination of the EIT-derived crossover PEEP trial
V_T_: Tidal volume
P_aw_: Airway pressure
ΔP_aw_: Driving pressure
P_aw,plat_: Airway plateau pressure
P_eso_: Esophageal pressure
ΔP_eso_: Change in esophageal pressure
P_eso,ee_: End-expiratory esophageal pressure
P_tp,ee_: End-expiratory transpulmonary pressure
C_stat_: Static respiratory system compliance
EELV: End-expiratory lung volume
EELV_breathgas_: EELV as measured from nitrogen washin/washout technique
EELV_CT_: EELV as determined from CT imaging
EELV_model_: EELV as predicted by the model
Meas: Values for measured data
Sim: Values for simulated data
F_left_: EIT signal fraction in the left thoracic half
F_dorsal_: EIT signal fraction in the dorsal thoracic half
CoV: Center of ventilation
CoV_rl_: Center of ventilation along the right-left axis
CoV_vd_: Center of ventilation along the ventral-dorsal axis Statistics
RMSE: Root mean square error
LoA: Limits of agreement
