# Supplementary material for: Predicting global and regional respiratory response in ARDS patients through individualized physics-based computational lung models: a prospective clinical pilot study
Source: Respir Res. 2026 Oct 17;27:414. doi: 10.1186/s12931-026-03949-z (PMC13685381; doi:10.1186/s12931-026-03949-z)
Supplement: Supplementary file 1 [file 12931_2026_3949_MOESM1_ESM.docx]

## SMART - Additional File 1

**Figure 1** Workflow of the computational modeling process. highlighting the point of use of clinical data (light blue), procedural steps (dark blue), and their sequence (arrows).
