# Supplementary material for: Predicting global and regional respiratory response in ARDS patients through individualized physics-based computational lung models: a prospective clinical pilot study
Source: Respir Res. 2026 Oct 17;27:414. doi: 10.1186/s12931-026-03949-z (PMC13685381; doi:10.1186/s12931-026-03949-z)
Supplement: Supplementary file 2 [file 12931_2026_3949_MOESM2_ESM.docx]

## SMART - Additional File 2

## EIT model and image reconstruction

EIT was employed to regionally validate the model’s ventilation predictions. It should be emphasized that the EIT measurements collected during patient ventilation were used solely for validation purposes and were not involved in the calibration of the digital twins.

Only some patients wore the EIT sensor belt during the CT scan, allowing for direct identification of electrode positions based on their visibility in the CT images.
For the remaining patients, electrode positions were estimated according to a standard clinical configuration, aligned along the costal arc based on the belt size and corresponding electrode spacing.

In this study, the EIT-based regional validation method described by Roth et al. (1) was implemented with modifications to accommodate the clinical measurement setup. The EIT validation procedure was implemented in the open source software package holoshed (2) which is based on the EIDORS toolbox and the GREIT reconstruction algorithm (3). The EIT model is based on a three-dimensional thorax model segmented from the patient CT scan and uses the finite element method for the electrical simulations. At its surface, the thorax model was equipped with 32 rectangular electrodes (10x40 mm) using a refined finite element mesh size 2 mm in contrast to 10 mm in the remaining domain).

Following Roth et al. (1,4) we mapped the locally simulated lung tissue strains, and hence air content in the mechanical model to the electrical resistivity of the lung tissue in the EIT model. Applying the same electrode stimulation pattern as in the clinical setup to this EIT model, we calculated virtual EIT electrode measurements that aim to mimic the behavior in the clinical measurements. Figure 1 shows the virtual EIT pipeline as a more detailed version of the virtual EIT module shown in Additional File 1.

As shown in Additional File 1, the virtual EIT module consists of two blocks ("Resistivity mapping" and "EIT simulation") and shares the EIT reconstruction block with the clinical EIT module. From the mechanical lung model, we get locally resolved values of the air, tissue, and fluid volume in the terminal units ($V_{air}$, $V_{tis}$, and $V_{liq}$). From these quantities, the corresponding filling factor $\mathrm{FF}$ and effective resistivity $\rho_{\mathrm{eff}}$ can be obtained via

$$\begin{aligned} FF&=\frac{V_{\mathrm{air}}}{V_{\mathrm{tis}}+V_{\mathrm{liq}}}, \\ \rho_{\mathrm{eff}}&=\frac{\tau}{\sigma_{\mathrm{alv}}}\cdot(FF+1), \end{aligned}$$

where $\tau=1.71$ is the mean tortuosity, and $\sigma_{\mathrm{alv}}=0.7284 \Omega^{-1}m^{-1}$  is the alveolar conductivity  (1,3). The effective resistivity $\rho_{\mathrm{eff}}$ is then mapped from the terminal units to tetrahedral elements of the EIT thorax mesh. Outside the lung regions, a constant conductivity of $\rho_{\mathrm{eff}}=4 \Omega m$ is considered.

The next big block is the actual forward simulation of the EIT problem, meaning calculating the voltage field $u$ given the resistivities. At all non-electrode surfaces, Neumann boundary conditions are set to zero, while the electrodes follow the Complete Electrode Model (CEM) described by Cheng et al. (5). As for the clinical EIT device, we use a 32-electrode belt, with a skip4 electrode pattern for stimulation, meaning there are 4 electrodes between two stimulating ones, and a stimulating current of I_stim_=5 mA. The voltage field u can then be described by the governing Laplace equation as

$$\begin{matrix} \nabla\cdot\left( \frac{1}{\rho}\nabla u \right)=0, \end{matrix}$$

where ρ is the resistivity distribution in the thorax mesh. As for the stimulation, the measuring electrodes are also paired with the skip4 pattern resulting in an array of 32 x 32 = 1024 measurements.

| Parameter | | | | | | Value | | |
| --- | --- | --- | --- | --- | --- | --- | --- | --- |
| Tortuosity $\tau$ | | | | | | 1.71 | | |
| Alveolar conductivity $\sigma_{\mathrm{alv}}$ | | | | | | 0.7284 $\frac{1}{\Omega m}$ | | |
| Electrode current $I_{\mathrm{stim}}$ | | | | | | 5 mA | | |
| Electrode pattern | | | | | | skip4 | | |
| Electrode size | | | | | | 10 x 40 mm | | |
| Hom. thorax resistivity $\rho_{\mathrm{thorax}}$ | | | | | | $4 \Omega m$ | | |
| Training points GREIT $N_{t}$ | | | | | | 1000 | | |
| Target size GREIT $s_{\mathrm{targ}}$ | | | | | | 0.05 | | |
| Weighting radius GREIT $r_{w}$ | | | | | | 0.15 | | |
| Boundary distance GREIT $s_{\mathrm{bound}}$ | | | | | | 2mm | | |
| Background conductivity ratio $\gamma_{\sigma}$ | | | | | | $0.3$ | | |
| Target density ratio $\gamma_{\rho}$ | | | | | | $3.0$ | | |
| Noise Figures NF are chosen according to EIT measurement quality: | | | | | | | | |
| Patient | 1 | 2 | 5 | 7 | 8 | | 9 | 10 |
| NF | 0.21 | 0.2 | 0.2 | 0.18 | 0.16 | | 0.16 | 0.16 |

**Table 1** Parameters of the virtual EIT module. The nomenclature is consistent with the work of Ludwig et al. (6)

**Figure 1** Detailed view of the virtual EIT module introduced in Appendix 1.

These electrode voltages from the virtual EIT forward model could directly be compared to the results of the clinical EIT measurements to assess the accuracy of the mechanical model predictions. However, as reconstructed EIT images are better to interpret than raw electrode voltages, we reconstructed the EIT images from both simulated and clinical EIT measurements using the reconstruction approach of Ludwig et al. that is based on the commonly used GREIT algorithm (3,6).

All necessary parameters for the virtual EIT module are summed up in Table 1. The nomenclature is consistent with the work of Ludwig et al. (6).

## References

1.	Roth CJ, Becher T, Frerichs I, Weiler N, Wall WA. Coupling of EIT with computational lung modeling for predicting patient-specific ventilatory responses. J Appl Physiol Bethesda Md 1985. 2017 Apr 1;122(4):855–67. doi:10.1152/japplphysiol.00236.2016 PubMed PMID: 27932671.

2.	GitLab [Internet]. 2024 [cited 2025 Nov 14]. Xenotaph3 / Holo Shed · GitLab. Available from: https://gitlab.com/xenotaph3/holoshed

3.	Adler A, Arnold JH, Bayford R, Borsic A, Brown B, Dixon P, et al. GREIT: a unified approach to 2D linear EIT reconstruction of lung images. Physiol Meas. 2009 Jun;30(6):S35. doi:10.1088/0967-3334/30/6/S03

4.	Roth CJ, Ehrl A, Becher T, Frerichs I, Schittny JC, Weiler N, et al. Correlation between alveolar ventilation and electrical properties of lung parenchyma. Physiol Meas. 2015 Jun;36(6):1211–26. doi:10.1088/0967-3334/36/6/1211 PubMed PMID: 26008964.

5.	Cheng KS, Isaacson D, Newell JC, Gisser DG. Electrode models for electric current computed tomography. IEEE Trans Biomed Eng. 1989 Sep;36(9):918–24. doi:10.1109/10.35300

6.	Ludwig M, Eichinger CM, Sablewski A, Frerichs I, Becher T, Wall WA. Anatomically informed GREIT reconstruction: improving EIT imaging for lung monitoring. Physiol Meas. 2026 Feb 16;47(2). doi:10.1088/1361-6579/ae4289 PubMed PMID: 41643319.
