# Supplementary material for: Predicting global and regional respiratory response in ARDS patients through individualized physics-based computational lung models: a prospective clinical pilot study
Source: Respir Res. 2026 Oct 17;27:414. doi: 10.1186/s12931-026-03949-z (PMC13685381; doi:10.1186/s12931-026-03949-z)
Supplement: Supplementary file 3 [file 12931_2026_3949_MOESM3_ESM.docx]

## SMART - Additional File 3

## **Figure 1** Assessment of Ventilation Distribution by Electrical Impedance Tomography

Assessment of ventilation distribution with EIT, using the fraction of ventilation in the dorsal and left region-of-interest (ROI) and by the geometrical center of ventilation (CoV). ROI fractions of the left and dorsal halves are referred to as F_dorsal_ and F_left_, respectively. For the CoV, two components were distinguished, namely the CoV_rl_ along the right-to-left axis and the CoV_vd_ along the ventral-to-dorsal axis. Values over $50 \%$ represent a ventilation that is shifted in the left and dorsal direction. Note that classical medical image conventions are used, where the left lung is on the right side of a transverse section.
