# Supplementary material for: Predicting global and regional respiratory response in ARDS patients through individualized physics-based computational lung models: a prospective clinical pilot study
Source: Respir Res. 2026 Oct 17;27:414. doi: 10.1186/s12931-026-03949-z (PMC13685381; doi:10.1186/s12931-026-03949-z)
Supplement: Supplementary file 4 [file 12931_2026_3949_MOESM4_ESM.docx]

## SMART - Additional File 4

## Additional model insights

## The physics-based modeling approach employed in this validation study has an important advantage over purely data-based approaches: internal model quantities are constrained by physical principles and can therefore be assigned a physical interpretation. Such quantities may provide useful clinical insight even if independent validation is not possible because no bedside measurement technique is available to provide a clinical reference. In this supplement, we present selected model-derived quantities that we expect to be of particular interest to the clinical community.

## A key parameter in the modeling approach of Geitner et al (1) is the factor of edema volume k_edema_ that defines the ratio of edema volume compared to the tissue volume. This is an input to the model that quantifies how much tissue is available for straining by occupying a part of the tissue, as it may be the case in the real lung with alveolar edema, and thus influences the pressure-volume curve of the model. It strongly affects the pressure-volume relationship of the model and is therefore identifiable during model calibration, within the assumptions of the model. Based on k_edema_, we derive the volume of edema V_edema_ and the volume of tissue V_tissue_ that are reported in Table 1 for the individual patients. These volumes remain constant throughout the simulation. Importantly, the model does not distinguish between alveolar and interstitial edema; V_edema_ should therefore be interpreted as an effective mechanical representation of edema-related fluid accumulation, assuming alveolar edema as a first approximation.

**Table 1:** Model calibrated lung tissue and edema volume. Values are model-derived estimates obtained during calibration and were not validated against an independent clinical reference

| **Patients** | **1** | **2** | **3** | **4** | **5** | **6** | **7** | **8** | **9** | **10** |
| --- | --- | --- | --- | --- | --- | --- | --- | --- | --- | --- |
| V_tissue_ (l) | 0.81 | 1.16 | 1.77 | 1.06 | 1.32 | 1.69 | 1.07 | 1.26 | 1.23 | 1.47 |
| V_edema_ (l) | 0.10 | 0.45 | 0.19 | 0.19 | 0.13 | 0.78 | 0.26 | 0.13 | 0.34 | 0.53 |

## Another insight derived from our computational model is the percentage of open lung volume. This quantity is obtained from the alveolar recruitment–derecruitment model developed by Geitner et al (1). Figure 1 presents the open lung volume for each patient case across all evaluated PEEP levels. This analysis allows us to assess the extent of recruitment associated with changes in PEEP. It is a clear extension to the capabilities of EIT as our model also allows us to quantify the absolute amount of non-aerated lung volume in contrast to the tidal information provided by EIT. A more detailed analysis could further characterize the effects of recruitment during individual ventilation maneuvers.

**Figure 1:** Mean end-expiratory open lung volume during PCV at different PEEP levels. Values are model predictions and were not validated against an independent clinical reference

**References:**

1.	Geitner CM, Köglmeier LJ, Frerichs I, Langguth P, Lindner M, Schädler D, et al. Pressure- and time-dependent alveolar recruitment/derecruitment in a spatially resolved patient-specific computational model for injured human lungs. Int J Numer Methods Biomed Eng. 2024;40(2):e3787. doi:10.1002/cnm.3787
