# Supplementary material for: Predicting global and regional respiratory response in ARDS patients through individualized physics-based computational lung models: a prospective clinical pilot study
Source: Respir Res. 2026 Oct 17;27:414. doi: 10.1186/s12931-026-03949-z (PMC13685381; doi:10.1186/s12931-026-03949-z)
Supplement: Supplementary file 5 [file 12931_2026_3949_MOESM5_ESM.docx]

## SMART - Additional File 5

## Data availability/exclusion

**Tidal Volume @ PCV:**

A total of 40 measurements were theoretically possible (10 patients × 4 PEEP levels). In Patient 5, the first low-flow maneuver at PEEP_init_ was aborted. Therefore, three measurements are missing, resulting in 37 data sets.

**Driving Pressure @ VCV:**

A VCV period was available only at two PEEP levels (PEEP_init_ and PEEP_EIT_). Although short VCV segments also appeared at PEEP_min_ and PEEP_max_, these occurred only in the context of hold maneuvers and maneuvers halving the driving pressure and did not contain stable periods of regular breaths. Thus, a maximum of 20 measurements was possible (10 patients × 2 PEEP levels). Measurements for Patient 5 are missing at both levels (see above), resulting in 18 data sets.

**Transpulmonary Pressure @ PCV:**

A total of 40 measurements were theoretically possible (10 patients × 4 PEEP levels). As above, Patient 5 had data only at PEEP_init_. In addition, Patient 4 was excluded due to highly unstable esophageal pressure signals. Thus, 3 measurements (Patient 5) plus all 4 PEEP levels for Patient 4 were excluded, resulting in 33 data sets.

**EIT (combined PCV and VCV plots):**

In total, 60 measurements were theoretically possible (40 PCV + 20 VCV), 6 for every patient. As mentioned above, measurements for Patient 5 were incomplete (only PCV at PEEP_init_) resulting in one datasets for Patient 5. No data were available for Patient 3, 4 and 6 due to unusable or missing EIT data (Patient 3: belt not worn/data lost; Patient 4 and 6: EIT data markedly affected by noise due to belt shift). These three patients were therefore excluded entirely. This results in 37 data sets (60 − 5 missing measurements for Patient 5 − 18 measurements for the three excluded patients).

**Data availability flowchart:**
