# Supplementary material for: Predicting global and regional respiratory response in ARDS patients through individualized physics-based computational lung models: a prospective clinical pilot study
Source: Respir Res. 2026 Oct 17;27:414. doi: 10.1186/s12931-026-03949-z (PMC13685381; doi:10.1186/s12931-026-03949-z)
Supplement: Supplementary file 6 [file 12931_2026_3949_MOESM6_ESM.docx]

## SMART - Additional File 6

| **Main characteristics of the study population** | | | | | | | | | | | | |
| --- | --- | --- | --- | --- | --- | --- | --- | --- | --- | --- | --- | --- |
| **Patient** | **1** | **2** | **3** | **4** | **5** | **6** | **7** | **8** | **9** | **10** | **Mean ± SD** | **Median (IQR)** |
| Age [years] | 51 | 81 | 75 | 59 | 67 | 72 | 63 | 25 | 56 | 57 | 60.6 ± 15.6 | 61.0 (56.25–70.75) |
| Body Height [cm] | 155 | 180 | 175 | 168 | 184 | 180 | 172 | 180 | 186 | 190 | 177.0 ± 10.1 | 180.0 (172.75–183.0) |
| Body Weight [kg] | 50 | 80 | 75 | 100 | 90 | 100 | 100 | 80 | 110 | 90 | 87.5 ± 17.2 | 90.0 (80.0–100.0) |
| BMI [kg/m²] | 20.8 | 24.7 | 24.5 | 35.4 | 26.6 | 30.9 | 33.8 | 24.7 | 31.8 | 24.9 | 27.8 ± 4.8 | 25.75 (24.7–31.575) |
| PF-Ratio [mmHg] | 220 | 176 | 240 | 120 | 283 | 83 | 120 | 220 | 93 | 180 | 173.5 ± 67.6 | 178.0 (120.0–220.0) |
| Tube size [ID] | 7.0 | 7.5 | 8.5 | 8.0 | 8.0 | 8.5 | 8.0 | 7.0 | 8.0 | 9.0 | 8.0 ± 0.6 | 8.0 (7.625–8.375) |
| ASA | 3 | 3 | 3 | 3 | 2 | 3 | 3 | 1 | 2 | 2 | 1 ASA1, 3 ASA2, 6 ASA3 | |
| Gender | f | m | m | m | m | m | f | f | m | m | 3:7 (female:male) | |
| PEEPinit [mbar] | 10 | 8 | 12 | 16 | 20 | 13 | 10 | 12 | 15 | 12 | 12.8 ± 3.5 | 12.0 (10.5–14.5) |
