# Supplementary material for: Predicting global and regional respiratory response in ARDS patients through individualized physics-based computational lung models: a prospective clinical pilot study
Source: Respir Res. 2026 Oct 17;27:414. doi: 10.1186/s12931-026-03949-z (PMC13685381; doi:10.1186/s12931-026-03949-z)
Supplement: Supplementary file 7 [file 12931_2026_3949_MOESM7_ESM.docx]

## SMART - Additional File 7

Comparison of Measured and Simulated Airway Pressure and Tidal Volume

**Figure 1** Comparison of measured and simulated airway pressure curves at PEEP_init_ and PEEP_EIT_ across all patients.

**Figure 2** Comparison of measured and simulated tidal volume curves at PEEP_init_ and PEEP_EIT_ across all patients.
