# Supplementary material for: Predicting global and regional respiratory response in ARDS patients through individualized physics-based computational lung models: a prospective clinical pilot study
Source: Respir Res. 2026 Oct 17;27:414. doi: 10.1186/s12931-026-03949-z (PMC13685381; doi:10.1186/s12931-026-03949-z)
Supplement: Supplementary file 8 [file 12931_2026_3949_MOESM8_ESM.docx]

## SMART - Additional File 8

| **Patient-level RMSE of model predictions for global and regional ventilation parameters.** | | | | | | | | | | |
| --- | --- | --- | --- | --- | --- | --- | --- | --- | --- | --- |
| Parameter [unit] | 1 | 2 | 3 | 4 | 5 | 6 | 7 | 8 | 9 | 10 |
| *V_T_ [l]* | 0.041  (n=4) | 0.073  (n=4) | 0.026  (n=4) | 0.022  (n=4) | 0.005  (n=1) | 0.039  (n=4) | 0.032  (n=4) | 0.051  (n=4) | 0.078  (n=4) | 0.080  (n=4) |
| *ΔP_aw_ [mbar]* | 1.222  (n=2) | 1.084  (n=2) | 1.565  (n=2) | 1.110  (n=2) |  | 1.620  (n=2) | 1.194  (n=2) | 2.035  (n=2) | 0.295  (n=2) | 1.118  (n=2) |
| *P_tp,ee_ [mbar]* | 3.496  (n=4) | 1.763  (n=4) | 0.927  (n=4) | – | 0.909  (n=1) | 2.249  (n=4) | 1.733  (n=4) | 1.325  (n=4) | 1.566  (n=4) | 1.034  (n=4) |
| *F_left_ [%]* | 4.751  (n=4) | 5.163  (n=4) | – | – | 1.085  (n=1) | – | 7.662  (n=4) | 3.213  (n=4) | 4.288  (n=4) | 5.643  (n=4) |
| *F_dorsal_ [%]* | 1.157  (n=4) | 5.453  (n=4) | – | – | 0.310  (n=1) | – | 12.623  (n=4) | 16.219  (n=4) | 9.376  (n=4) | 18.415  (n=4) |
| *CoV_rl_ [–]* | 0.026  (n=4) | 0.031  (n=4) | – | – | 0.003  (n=1) | – | 0.035  (n=4) | 0.010  (n=4) | 0.018  (n=4) | 0.031  (n=4) |
| *CoV_vd_ [–]* | 0.005  (n=4) | 0.029  (n=4) | – | – | 0.002  (n=1) | – | 0.058  (n=4) | 0.069  (n=4) | 0.047  (n=4) | 0.110  (n=4) |
