# Supplementary material for: Predicting global and regional respiratory response in ARDS patients through individualized physics-based computational lung models: a prospective clinical pilot study
Source: Respir Res. 2026 Oct 17;27:414. doi: 10.1186/s12931-026-03949-z (PMC13685381; doi:10.1186/s12931-026-03949-z)
Supplement: Supplementary file 9 [file 12931_2026_3949_MOESM9_ESM.docx]

## SMART - Additional File 9

## Mismatch in ventral-dorsal ventilation as determined by EIT

## In our analysis of the predictive capacity of the computational model, we observed a clear difference in error magnitude between the ventral-dorsal and right-left directions. Specifically, Table 4 of the main part of this study reports an RMSE 4.980% in F_left_, compared with 12.139% in F_dorsal_. To further investigate this discrepancy, we provide additional contextual data in this supplement, enabling a more informed assessment of the model's performance.

**Figure 1:** Absolute errors in the fractions of left and dorsal ventilation over the PEEP levels

Figure 1 shows that the difference in error magnitude between the two axes is consistently observed across all evaluated PEEP levels. Furthermore, analysis of F_dorsal_ reveals a clear tendency across patients for the smallest error magnitude to occur at PEEP_max_. This finding suggests that the observed ventrodorsal mismatch is unlikely to originate from limitations of the EIT based comparison itself, but may instead be related to assumptions within the mechanical model.

To further contextualize these findings with respect to the overall patient characteristics, we investigated the potential influence of BMI, as shown in Figure 2. Because of the small sample size, with only six to seven measurements available depending on the comparison, and the considerable heterogeneity among patients, these observations should be regarded as exploratory and not used to draw firm conclusions. The data shows a positive association between BMI and ventrodorsal error at PEEP_init_ (r = 0.342, p = 0.453), PEEP_min_ (r = 0.283, p = 0.587), and PEEP_EIT_ (r = 0.361, p = 0.482). In contrast, no corresponding positive association was observed at PEEP_max_ (r = -0.190, p = 0.718). These findings are exploratory and are not statistically significant. We could not find a clear association of the ventral-dorsal mismatch and the ARDS severity.

As reported in Table 4 in the main manuscript, the model exhibits a systematic bias in the regional ventilation distribution by overestimating the dorsal ventilation fraction. We hypothesize that the model may underestimate the pleural pressure in the dorsal region because it does not account for the influence of the abdominal organs. In the current modeling approach, the pressure at the dorsal boundary of the lungs depends only on lung weight and does not incorporate the contribution of abdominal weight. This may lead to an overestimation of transmural pressure in the dorsal region and, consequently, an overprediction of dorsal ventilation. At PEEP_max_, this effect is reduced as the influence of the boundary conditions decreases with increasing baseline pressure.
However, the overprediction of dorsal ventilation is unlikely to be attributable to a single mechanism and therefore requires further investigation and more advanced modeling approaches. The pleural boundary condition results from a complex interaction between the various tissues and organs surrounding the lungs. In addition, the nonlinear, sigmoidal pressure–volume relationship of alveolar tissue may contribute to regional differences in ventilation.

## **Figure 2:** Exploratory association of ventral-dorsal mismatch and BMI
